# A case study for efficient management of high throughput primary lab data

**DOI:** 10.1186/1756-0500-4-413

**Published:** 2011-10-17

**Authors:** Christian Colmsee, Steffen Flemming, Matthias Klapperstück, Matthias Lange, Uwe Scholz

**Affiliations:** 1 Leibniz Institute of Plant Genetics and Crop Plant Research (IPK), Corrensstr. 3, 06466 Gatersleben, Germany

## Abstract

**Background:**

In modern life science research it is very important to have an efficient management of high throughput primary lab data. To realise such an efficient management, four main aspects have to be handled: (I) long term storage, (II) security, (III) upload and (IV) retrieval.

**Findings:**

In this paper we define central requirements for a primary lab data management and discuss aspects of best practices to realise these requirements. As a proof of concept, we introduce a pipeline that has been implemented in order to manage primary lab data at the Leibniz Institute of Plant Genetics and Crop Plant Research (IPK). It comprises: (I) a data storage implementation including a Hierarchical Storage Management system, a relational Oracle Database Management System and a BFiler package to store primary lab data and their meta information, (II) the Virtual Private Database (VPD) implementation for the realisation of data security and the LIMS Light application to (III) upload and (IV) retrieve stored primary lab data.

**Conclusions:**

With the LIMS Light system we have developed a primary data management system which provides an efficient storage system with a Hierarchical Storage Management System and an Oracle relational database. With our VPD Access Control Method we can guarantee the security of the stored primary data. Furthermore the system provides high performance upload and download and efficient retrieval of data.

## Background

**Figure 1:**
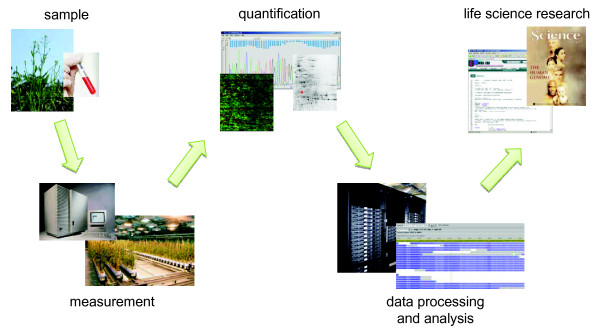
**Primary data workflow**. General primary data workflow in life sciences describing how primary data is generated. Experiments comprise different samples; based on experiment and sample, several measurements are performed. After quantification there is a step of data processing and analysis which results in life science research.

Modern life sciences research is depending on powerful IT-infrastructure. The process of gaining knowledge is tightly coupled with the data producer, bioinformatics tools, the structured data storage and long term archiving. In this context, Laboratory Information Management Systems (LIMS) are getting an increased focus in life sciences. Examples for LIMS implementations are presented in [1,2] and [3]. The most common definition of a LIMS can be summarised as follows: LIMS is a computer software that is used in the laboratory for the management of samples, laboratory users, equipment, standards and other laboratory functions, such as invoicing, plate management and work flow automation. However, an important basis of all laboratory processes is the *primary data*. Primary data is read-only raw data that comes directly or indirectly from molecular biological analysis devices. The data must be available for all kinds of subsequent analysis and result interpretation (see Figure 1).

The data domain is manifold and ranges from DNA mapping and sequences, gene expression, proteomics to phenotyping. Over the past several decades, many of the life sciences have been transformed into high-throughput areas of study [4]. In a number of cases, the rate at which data can now be generated has increased by several orders of magnitude. Such dramatic expansions in data throughput have largely been enabled by engineering innovation, e.g. hardware advancements and automation. In particular, laboratory tasks that were once performed manually are now carried out by robotic fixtures. The growing trend towards automation continues to drive the urgent need for proper IT support [5]. Here, we use the acronym IT to name those infrastructure services that (a) process and analyse data, (b) organise and store data and provide structured data handling capability and (c) support the reporting, editing, arrangement and visualisation of data. The aim is to reflect the biologists data ⇒ information ⇒ knowledge paradigm [6] and to meet the requirements of high-throughput data analysis and the resulting data volume of petabytes [7].

The Leibniz Institute of Plant Genetics and Crop Plant Research (IPK), Germany, is a research centre, which primarily applies the concepts and technologies of modern biology to crop plants and hosts the German central *ex situ *gene bank. There are many scientific fields at the IPK producing high throughput data, such as plant phenotyping (http://www.lemnatec.com) and 454-sequencing [8]. One recurring task for bioinformatics at the IPK is the implementation of databases and information systems. To handle, manage and secure a huge amount of data properly, it is always a great advantage to have a well trained bioinformatics and IT staff which takes care for it and assumes responsibility for the valuable electronic information. We learned which design principles and implementation techniques are useful and which have to be avoided. Subsequently, we abstracted a best practice and proven implementation concept as experiences from several bioinformatics projects: SEMEDA (Semantic Meta Database) for providing semantically integrated access to databases and to edit and maintain ontologies and controlled vocabularies [9], MetaCrop, a manually curated repository of high quality information about the metabolism of crop plants [10] and an integration and analysis pipeline for systems biology in crop plant metabolism [11]. In this paper we summarise these experiences into best practice for primary data management. First, we will present the requirements of primary data management. Afterwards, we will give an overview to different solutions of primary data management. Furthermore, we will present our results and we will last but not least list our used methods.

### The major aspects of primary data management

There are four major aspects about the handling of primary data. Today, automated techniques produces a large amount of data. It is necessary to find solutions for large storage capacity and efficient backup mechanism in a preferably affordable way. Especially during the life span of a project the data access has to be restricted to project related users. Finding a way to manage the access rights for users and even groups within the primary data management will be another important aspect. One most important fact about primary data management is the performance of storing files and an efficient retrieval of the stored data. The third main aspect would be to realise such an efficient system. The last important aspect is the usability of the system. The user interface has to be simple and intuitive for the different biological groups using the system.

### The central requirements of primary data management

**Figure 2:**
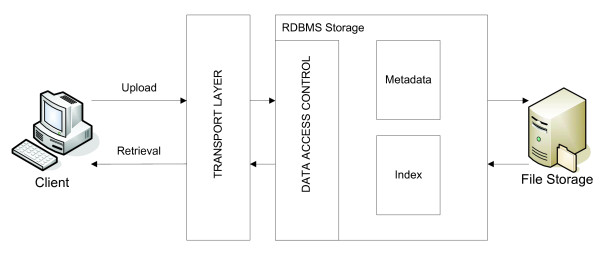
**universal system architecture or primary lab data management**. Architecture to realise a system for primary lab data management - The architecture includes components for upload and retrieval of primary lab data, a transport layer to transfer the data from the client side to the server side, a RDBMS storage component comprising a data access control, a metadata storage and index for fast data retrieval, and a file storage component to store the primary lab data itself.

Figure 2 illustrates the architecture that has to be realised for a primary lab data management system. There have to be components for upload and retrieval of this data. Next, the data has to be secured so that only authorised users can access specific data. The last important fact is the storage. The data has to be stored efficiently and the metadata has to be available for data retrieval.

Referring to the four major aspects of primary data management and the architecture in Figure 2 there can be derived several requirements for managing high throughput primary lab data:

• data storage:

- storage of metadata from files for later retrieval

- central storage of primary lab data for decentralised computation

- versioning of data

• data security:

- upload and retrieval of data for authorised users

- invariability of data has to be guaranteed

• data upload and retrieval:

- fast upload of large amount of data

- automation of data upload (including automated tagging of files)

- fast and efficient retrieval of stored files

Best practices for the above listed requirements are presented in the following sections.

### Solutions for primary data management

A major aspect in scientific research is the citation of data for publications. Anderson et al. [12] show, that there is a increasing number of publications containing supplementary material and that data has to be long-term archived. But often data is not longer available, especially for elder publications. To make data citable a persistent identifier linked with the data is needed. Such identifier is provided by DOI (Digital Object Identifier, http://www.doi.org). One system working with DOIs is the Pangaea system (http://www.pangaea.de/), a publishing network for geoscientific and environment data. It uses TIB, the German National Library of science and technology, which provides services for DOI registration. TIB is a member of DataCite, an organisation with currently 15 members and 4 associate members, which has the main goal to guarantee easy access to scientific data (http://www.datacite.org). Another motivation is shown by Piwowar et al. [13]. There is a correlation between the publication of research data and the number of citations. Publications with shared data are more often cited than publications without any publicly available data.

The creation of central data repositories is a further task of scientific research. One example of such a repository is GabiPD [14]. GabiPD stores data from high throughput experiments of different plant species and further provides methods to analyse and visualise the stored data. The online archive PsychData [15] is an open access central repository which provides long term documented data over all aspects of psychology. With this archive the scientific community has a platform to store and to exchange their data with other scientists, which leads to a better collaborative scientific usability of the data. Another system to store scientific data is the LabKey Server [16]. The main advantage of this platform is the ability to integrate, analyse and visualise data from diverse data sources, which is very useful in large research consortia. For digital long term storage, in Germany the nestor organisation was founded (http://www.longtermpreservation.de). It provides a guideline of standardisation activities regarding persistent identifiers, metadata, file formats, certification, reference models and records management. Nestor is also partner in the European community APA (alliance for permanent access, http://www.alliancepermanentaccess.org/).

## Results and discussion

### The LIMS Light system

**Figure 3:**
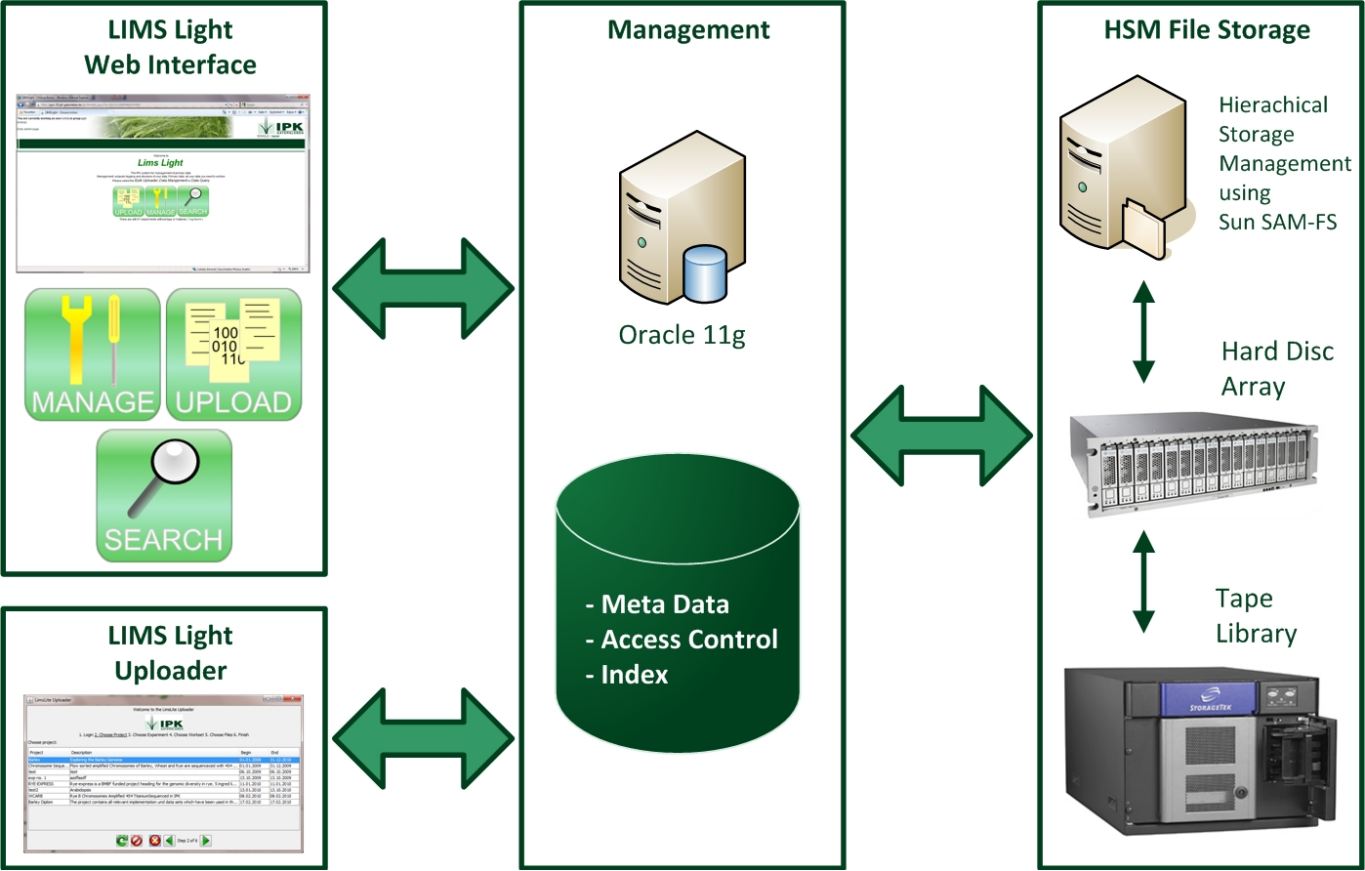
**IPK system architecture or primary lab data management**. Primary lab data management pipeline comprising the client with LIMS Light Uploader and APEX web application, the Database Server with the BFiler Database package and the LIMS Light metadata scheme, the HSM file storage and the Application Server with the Download Servlet.

To handle the four major aspects of primary data management and to realise the specified requirements we have implemented a pipeline that is shown in Figure 3. Within the LIMS Light Web Interface, developed with the Oracle Application Express (http://apex.oracle.com) technology, the users have the possibility to manage, upload and retrieve the primary data. The upload itself is performed by a Java web start application called LIMS Light Uploader. The meta data is stored in an Oracle 11 g database. Additionally, the search performance is improved by the usage of Oracle Index and the data access control is guaranteed with Oracle's Virtual Private Database (VPD) technology. The primary data itself is stored on a Hierarchical Storage Management (HSM) System using SAM-FS, which is a two layer system comprising a hard disc array for fast data access and a tape library for cheap long-term storage.

### Efficient storage system

We have implemented a system that balances between long-term storage and fast data access by using the HSM technology. The hard disc layer guarantees a fast data access and with the tape layer we have a cheap storage media in relation to hard disks. The HSM is configured in a specific way to fulfil our storage policy. Every twenty minutes new files are copied to two tapes. Regularly the second copy is removed from the library and is stored in an external shelf. Each file is kept on the disc cache for at least five minutes. If the disc space is fallen below five percent of capacity the oldest files are released first. But the first 16K of every file is kept on the disc. Additionally, we have an efficient way to backup our data. Instead of storing the data as BLOBs in the database and with those using expensive hard disks we use Oracle BFILEs to link the metadata with the files stored on the HSM. Thus the metadata backup within the database would be very efficient. Furthermore, the HSM supports an automatic backup from all files because they are stored twice on the tape layer.

### Proven data access control method

By using the row level security concept, we support fine-grained data access control that guarantees data privacy for all stored primary data. The row level security concept is an aproach which is used in various database systems. Commercial DBMS offer row level security implementations like DB2 with Label Based Access Control (LBAC), as well as open source systems like Security Enhanced PostgreSQL (SE-PostgreSQL). The advantage of this concept is that a project manager has an application and database query language independent method to secure operations and table rows at the database level. This is achieved by the automated modification of database queries by the database system itself. The benefit is the possibility of granting direct SQL access without injuring authorisation policies. With this system we are able to manage all users and workgroups from the IPK and can also grant access for external project partners to the LIMS Light system without coding the authorisation policies in a middleware or frontend.

### Efficient upload and retrieval of primary data

**Figure 4:**
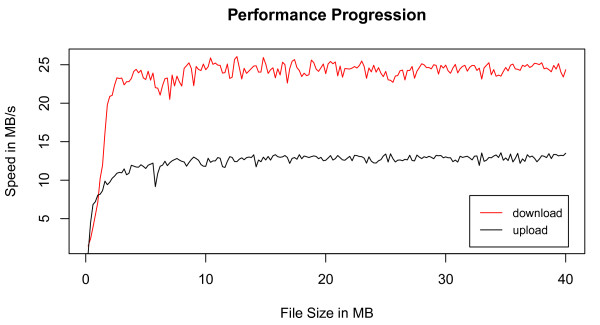
**LIMS Light performance progression**. This Figure shows the upload and download performance of LIMS Light. The low performance on small files is the result of a large metadata overhead.

**Figure 5:**
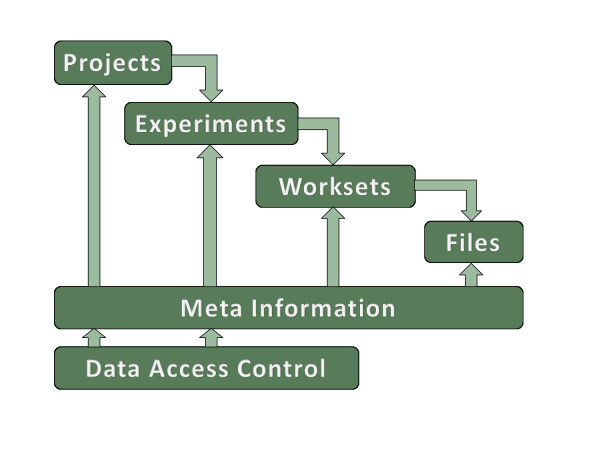
**LIMS Light workflow**. The LIMS Light workflow has a hierarchical structure. The files are located in worksets which are related to experiments. These experiments are connected to a specific project. All data is described by meta information. Furthermore, the projects and experiments are protected by a data access control.

With the LIMS Light Uploader we can upload our primary data with a speed of approximately 13 MB/s. Further, we reach a download speed of approximately 24 MB/s. The used benchmark environment was a 1GBit Ethernet network. Figure 4 illustrates a problem with large metadata overhead, when uploading or downloading small files. But with files larger than approximately 3 MB we reach a high performance. The upload of large files is limited mainly by the configured size of temporary tablespace in the database which is practically unlimited and can be extended on demand. This is only constrained by the quantity of concurrent users. The maximum download speed shows that we reach nearly maxiumum network speed. The significant lower upload speed is caused by the greater amount of necessary metadata management actions. Handling small files is not a matter of data rate but a matter of the number of roundtrips and their latency caused by a high amount of API calls through the whole calling hierarchy. The Uploader supports an automatic file tagging by using configuration files comprising the file name, file tag and file description. Uploading single files is possible as well as the upload of multiple files. The directory structure of multiple file uploads is mapped to our easy and innovative LIMS Light workflow, which can be seen at Figure 5. The fast retrieval of data stored in LIMS Light is efficient as well by using Oracle TEXT index. The users can search for names and descriptions on each level of the workflow and also for ontology terms provided by the Ontology Lookup Service (OLS) [17].

### The LIMS Light data

In LIMS Light we are able to store primary data of many different data domains. Currently there are stored more than 30.000 files in the system with a total data volume of approximately 900 GB. With 307 GB, sequence data consumes the most storage space followed by image data (145 GB) and textual data (135 GB).

### A functional overview of LIMS Light

As mentioned before, LIMS Light has three main components, the management, the upload, and the retrieval of data. With the management component the user can create and modify all objects defined in the workflow. When creating a project the user can specify the project name and description, the responsible person for the project and the project start and end timepoint. Additionally, it can be defined which users and groups will have access to the project. The creation of an experiment includes information about the experiment name, description and date. The user can also assign material and associate ontologies to the experiment. The access rights for users and groups can be specified here as well as in the projects part. In the workset view the user can create new worksets including their description. Within the worksets, single or multiple files can be uploaded with the LIMS Light Uploader. The uploader is a wizard based tool which comprises the selection of project, experiment and workset. Projects and experimentsd can only be selected, if access rights were granted to the user. Inside the selected workset the user can select files and directories for upload. The retrieval of data has two parts, a search component and a download tool. With the search function the user can search for specific data containing a specific project, experiment, workset or filename. Additionally the user can search for data comprising a specific ontology, material or description. The Downloader is able to retrieve the data stored in the LIMS Light system. The user can decide wether a single file or multiple files or even whole worksets including subworksets should be downloaded.

### LIMS Light usecase

The web interface supports the LIMS Light workflow, thus enabling the user to manage projects, experiments, worksets and files. This workflow makes it easy for all the different biological groups to upload their primary data. A user with sequence data can use it as well as a user storing image data. To show the principles of LIMS Light in a more practical way we have uploaded a usecase for public access (http://limslight.ipk-gatersleben.de (user: limslight_public, passwort: limslight_public)).

**Figure 6:**
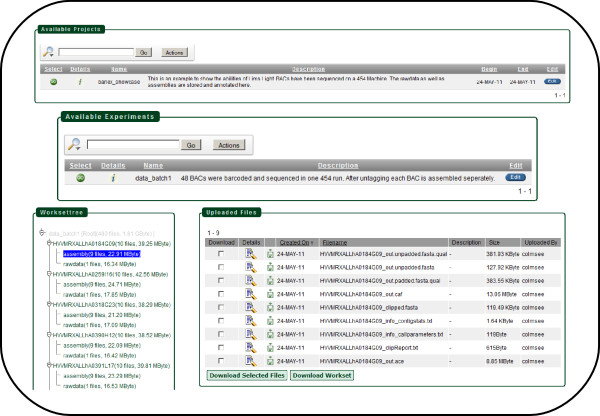
**LIMS Light usecase screenshot**. In this Figure the usecase barlex_usecase is presented by several screenshots comprising the project view, the experiment view, the workset view as well as the uploaded files.

The usecase_*barlex usecase *comprises 48 BACs that have been sequenced on a 454 sequencing machine [8]. The raw data is stored as well as the assembly data. Within the LIMS Light workflow barlex_usecase is the project. The experiment data_batch1 is representing set one of the BAC sequencing. The set two, mentioned in the publication, could be stored here as a second experiment. The workset view shows one workset for each BAC. Within this BAC worksets there are two subworksets for the raw data and the assembly files. The Figure 6 shows the screenshots for the project, experiment and workset view of the barlex_usecase. To show the functionality of the LIMS Light search every raw file contains a description. When for example searching for organism *Hordeum vulgare *the BAC sequence files will be listed within the search result.

## Conclusions

With the LIMS Light System we developed a primary data management system which provides an efficient storage system with a Hierarchical Storage Management System and an Oracle relational database. With our VPD Access Control Method we can guarantee the security of the stored primary data. We further developed a system which provides a high performance upload and download and an efficient retrieval of the data. With those first steps we are now able to proceed for the next step which is the integration of data citation so that scientists, using the LIMS Light system, can cite their primary data within their publications.

## Methods

### Primary lab data storage

For handling the primary lab data two kinds of data have to be stored. These are on one hand the data files themselves and on the other hand the metadata. The files are stored on a file system (also recommended by [18]). We used the hierarchical storage management (HSM) system SAM-FS (version 4.6.5) running under Sun Solaris 10.5. The meta information (e.g. taxonomy data, experiment conditions, genotypes, etc.) is stored in the relational database system Oracle (Oracle Enterprise Server 11 g version 11.2.0.2.0 - 64bit). Additionally, the references to the files are managed within the database together with the metadata. Therefore, the concept of Oracle BFiles as a special data type within the relational tables is used.

### Data security

Data security as an important aspect of primary lab data management is realised application independent. The system has to guarantee, that data is only accessible for specific users and groups. We used the row based concept of data protection called *RLS (Row Level Security)*. It is a fine-grained access control to limit the access at the row level for different operations (select, insert, update, delete) by defining a specific policy [19]. RLS is implemented in Oracle as Virtual Private Database (VPD). The principle is to transparently add a where-clause to every statement issued against the data via SQL. This additional predicate is provided by a user defined database function, which is running in a privileged mode and can use metadata for decision. The management of specific access rights is stored in a separate schema of the used Oracle Database.

### Data retrieval and upload

To fulfil the performance requirements for uploading primary lab data a combined approach is used. With the Oracle Application Express Technology (version 4.0.1.00.03) a web based user interface was developed. This interface supports the management of the metadata (e.g. file name, file type, author, file description, etc.). A connection to the Ontology Lookup Service (OLS) [17] is integrated. This helps to classify the origin of a file with controlled vocabularies. Furthermore the data retrieval is supported by the web based user interface. The file upload is handled by a Java Webstart Application (version 1.6), which can be started from the web based user interface to support a batch upload of selected files and/or folders.

## Availability and requirements

The source codes of LIMS Light are available at http://dx.doi.org/10.5447/IPK/2011/0

**Project name: **LIMS Light

**Project homepage: **http://limslight.ipk-gatersleben.de (user: limslight public, password: limslight_public, read only access on a test dataset)

**Operating system: **platform independent

**Programming language: **Java, PL/SQL

**Other requirements: **Oracle, Java 1.6

**License: **GPL 2.0

**Any restrictions to use by non-academics: **Please contact the authors before using the system.

## Competing interests

The authors declare that they have no competing interests.

## Authors' contributions

ML and CC drafted the manuscript. CC and ML implemented the uploader tool. CC and MK implemented the Web Frontend. SF implemented the BFiler. ML and US supervised the project. All did proof-reading and manuscript editing. All authors read and approved the final manuscript.
